# Immunomodulatory Therapy of Visceral Leishmaniasis in Human Immunodeficiency Virus-Coinfected Patients

**DOI:** 10.3389/fimmu.2017.01943

**Published:** 2018-01-12

**Authors:** Wim Adriaensen, Thomas P. C. Dorlo, Guido Vanham, Luc Kestens, Paul M. Kaye, Johan van Griensven

**Affiliations:** ^1^Unit of HIV and Neglected Tropical Diseases, Department of Clinical Sciences, Institute of Tropical Medicine, Antwerp, Belgium; ^2^Department of Pharmacy and Pharmacology, Antoni van Leeuwenhoek Hospital, Netherlands Cancer Institute, Amsterdam, Netherlands; ^3^Unit of Virology, Department of Biomedical Sciences, Institute of Tropical Medicine, Antwerp, Belgium; ^4^Unit of Immunology, Department of Biomedical Sciences, Institute of Tropical Medicine, Antwerp, Belgium; ^5^Centre for Immunology and Infection, Department of Biology, Hull York Medical School, University of York, Heslington, York, United Kingdom

**Keywords:** visceral leishmaniasis, kala-azar, human immunodeficiency virus, immunotherapy, immunomodulation, coinfection, immunity, vaccination

## Abstract

Patients with visceral leishmaniasis (VL)–human immunodeficiency virus (HIV) coinfection experience increased drug toxicity and treatment failure rates compared to VL patients, with more frequent VL relapse and death. In the era of VL elimination strategies, HIV coinfection is progressively becoming a key challenge, because HIV-coinfected patients respond poorly to conventional VL treatment and play an important role in parasite transmission. With limited chemotherapeutic options and a paucity of novel anti-parasitic drugs, new interventions that target host immunity may offer an effective alternative. In this review, we first summarize current views on how VL immunopathology is significantly affected by HIV coinfection. We then review current clinical and promising preclinical immunomodulatory interventions in the field of VL and discuss how these may operate in the context of a concurrent HIV infection. Caveats are formulated as these interventions may unpredictably impact the delicate balance between boosting of beneficial VL-specific responses and deleterious immune activation/hyperinflammation, activation of latent provirus or increased HIV-susceptibility of target cells. Evidence is lacking to prioritize a target molecule and a more detailed account of the immunological status induced by the coinfection as well as surrogate markers of cure and protection are still required. We do, however, argue that virologically suppressed VL patients with a recovered immune system, in whom effective antiretroviral therapy alone is not able to restore protective immunity, can be considered a relevant target group for an immunomodulatory intervention. Finally, we provide perspectives on the translation of novel theories on synergistic immune cell cross-talk into an effective treatment strategy for VL–HIV-coinfected patients.

## Introduction

Visceral leishmaniasis (VL), also called kala-azar, is a vector-borne protozoan infection caused by species of the *Leishmania donovani* complex, which mainly targets tissue macrophages of systemic organs, such as spleen, liver, and bone marrow (1). Characteristics of the disease include chronic fever, hepatosplenomegaly, and pancytopenia (1). Untreated, overt disease is universally lethal (1). Zoonotic VL, with dogs as the main reservoir, is mainly prevalent in the Mediterranean basin and in South America, and is caused by *Leishmania infantum*. Anthroponotic VL is prevalent on the Indian subcontinent and in East Africa and is typically caused by *L. donovani* (2). According to the recent World Health Organization (WHO) report, VL is endemic in 75 countries with an estimated 50,000–90,000 new cases occurring each year (3). Ninety percent of the global disease burden occurs in just six countries: India, Bangladesh, Sudan, South Sudan, Brazil, and Ethiopia (3).

Chemotherapy is currently the sole form of treatment in clinical practice. The pentavalent antimonial (Sb^V^) compounds [sodium stibogluconate (SSG) commercialized as Pentostam^®^; meglumine antimoniate commercialized as Glucantime^®^] have been the cornerstone of first-line treatment of VL over the last 70 years. However, these compounds are far from optimal due to severe toxicity and the emergence of antimonial resistance on the Indian subcontinent (1, 4). Newer drugs that are increasingly used include paromomycin, miltefosine, pentamidine, and conventional and liposomal amphotericin B. All these drugs have several important disadvantages as shown in Table 1. While various combination therapy regimens designed to overcome some of the shortcomings are highly efficacious in India, disappointing findings on some combination regimens have been recently reported in East Africa (5–10). As of today, no comparative studies have been conducted to explain this geographical difference, but parasite genetic diversity and host immune phenotypes are assumed as key factors. Novel chemotherapeutic drugs are in the initial development pipeline and are, therefore, unlikely to be widely available within the next few years. Nevertheless, over 90–95% of immunocompetent patients display a good clinical response to currently recommended conventional treatment regimens, with treatment unresponsiveness, death or severe toxicity observed in less than 5–10% of patients (11). Less than 5% of immunocompetent individuals who initially cure develop a relapse, most commonly within 6–12 months after treatment (5). Treatment outcomes, however, vary substantially between different geographic regions and depend on the drug(s) used, drug exposure, parasite susceptibility to the drug, severity of disease, host immunity, and the presence of coinfections (11–13).

**Table 1:** The main drugs currently used for treatment of visceral leishmaniasis (VL), adapted from Ref. (5).

Drug	Toxicity	Main limitations
Pentavalent antimonials (Sb^V^)	Frequent, potentially severe – Pancreatitis – Cardiotoxicity – Nephrotoxicity – Hepatotoxicity	Toxicity (high mortality in human immunodeficiency virus (HIV)-coinfected African patients) Painful injection (im) Length of treatment Resistance in India

Conventional amphotericin B deoxycholate	Frequent infusion-related reactions – Nephrotoxicity – Hypokalemia	Lengthy hospitalization (in-patient care) Slow iv infusion Nephrotoxicity

Liposomal amphotericin B (AmBisome)	Uncommon and mild – Nephrotoxicity (limited)	High price Slow iv infusion Heat instability (<25°C) Accessibility Single dose not effective in East Africa

Miltefosine	Common, usually mild and transient – Gastrointestinal – Hepatotoxicity	Relatively limited efficacy data in East Africa Possibly teratogenic Potential for resistance^a^ Patient compliance (oral drug) High price

Paromomycin sulfate (aminosidine)	Common – Ototoxicity – Nephrotoxicity – Hepatotoxicity	Toxicity (Oto- and nephrotoxicity) Resistance readily obtained in lab isolates Efficacy variable between and within regions (less in Sudan)

Pentamidine	Common – Gastrointestinal – Cardiotoxicity – Pancreatitis – (Ir)reversible diabetes mellitus	Low efficacy Toxicity (diabetes, renal failure) Length of treatment

*^a^Due to long half-life + low genetic barrier (resistance readily obtained in lab isolates)*.

*iv, intravenous injection; im, intramuscular injection*.

### Emerging Challenge of VL–HIV Coinfection

Human immunodeficiency virus (HIV) has been identified as one of the emerging challenges facing the control of VL (14). The immunological status of HIV-infected patients is particularly favorable for the multiplication of *Leishmania* parasites. HIV coinfection substantially increases the risk of progression from asymptomatic *Leishmania* infection to active disease (14, 15). On the other hand, VL accelerates HIV disease progression towards acquired immunodeficiency syndrome (AIDS) and could induce expression of latent proviruses (14). HIV has fueled the re-emergence of VL in Southern Europe and Brazil, where up to 70% of VL cases are associated with HIV infection (7). The problem is currently particularly severe in areas such as Northern Ethiopia, where up to 30% of all VL patients are coinfected with HIV (16). Since 2001, 35 countries have reported between 2 and 30% of VL cases as coinfected with HIV, but these percentages are most probably underestimations (14). Because the disease affects the most poor and most neglected patients within an already neglected disease population, under-reporting in most endemic areas is common due to a lack of facilities to diagnose one or both of the diseases and to poor reporting systems. Importantly, VL–HIV-coinfected patients are also often considered super-spreaders of VL and, thus, pose a major threat to current elimination strategies (17).

Since 1996, combined antiretroviral treatment (cART), comprising three antiretroviral drugs, constitutes the cornerstone of HIV treatment. The treatment options continue to expand with new drugs and co-formulations; by the end of 2016, there were 40 antiretroviral drugs from six different classes approved by the Food and Drug Administration. In most resource-constrained settings, the standardized WHO guidelines are used for ART, which currently recommends a combination of tenofovir, lamivudine, and efavirenz as first-line treatment. WHO recommended first-line regimens have been found highly effective in resource-constrained settings (18). The main aim of cART is sustainable suppression of HIV replication, and with good adherence, this can generally be achieved, leading to a close to normal life expectancy (19).

Visceral leishmaniasis is one of the AIDS-defining conditions, requiring anti-leishmanial treatment and cART irrespective of CD4^+^ T cell count (7). Although there are limited *in vitro* data suggesting that HIV-1 protease inhibitors and possibly some other antiretroviral drugs might directly exert inhibitory effects on *Leishmania*, there is insufficient evidence for their clinical use against VL, and standard ART regimens are currently recommended in VL–HIV coinfection (5). In low income countries, this is provided by standardized first- and second-line regimens in a public health approach (20, 21).

Increased toxicity and parasitologically confirmed treatment failures (up to 30%) were observed in VL–HIV-coinfected patients treated with Sb^V^, with case fatality rates up to 24% (14, 16, 22). While liposomal amphotericin B was consistently found to have excellent tolerability, VL cure rates in HIV-coinfected individuals have been rather disappointing in East Africa. For example, at a total dose of 30 mg/kg, around 16% of primary VL and 56% of VL relapse cases demonstrate parasitological failure in northern Ethiopia (16). WHO now proposes a total dose of 40 mg/kg (7, 23, 24). Experience with miltefosine in VL–HIV coinfection is limited, but suggests moderate efficacy and an acceptable toxicity profile (22, 25–28). To date, only one clinical trial in HIV-coinfected patients has been conducted with miltefosine, with 18% of patients displaying initial parasitological treatment failure and 25% relapsing, although deaths were excluded (22). The role of combination therapy in VL–HIV coinfection is currently under exploration in clinical trials in India and East Africa.

While in Europe widespread use of cART has resulted in a pronounced (i.e., 60%) reduction in the incidence of VL–HIV coinfection, relapse in coinfected subjects remains substantial at up to 60% after 1 year (14, 29, 30) and secondary prophylaxis has only a partial effect (31). In a pentamidine secondary prophylaxis trial in Ethiopia, the relapse-free survival rate at 2 years was only 58.3% (32). Even with access to all current chemotherapies, the prognosis in VL–HIV coinfection remains dire. Currently, it is believed that VL can only be effectively treated in HIV patients before profound immune deficiency has developed.

Visceral leishmaniasis–HIV coinfection has a number of unique clinical and immunological features. In contrast to many other HIV-associated opportunistic infections, CD4^+^ T cell reconstitution is severely delayed (even if virological suppression is reached) and the immune reconstitution inflammatory syndrome to a *Leishmania* infection after initiation of cART appears relatively rare, indicating a persistent suppression of host immunity (33, 34). Atypical clinical presentations can occur and amastigotes have been detected in tissues such as the intestine, where parasites are mostly undetectable in the immunocompetent host (14, 35). After clinical remission, parasitemia also appears to persist, at least intermittently (36). A chronic/intermittent course of VL lasting several years has been described, labeled as “active chronic visceral leishmaniasis” (36). Consequently, HIV-infected patients will develop multiple VL relapses and often become progressively more difficult to treat, ultimately leading to a stage of complete treatment unresponsiveness. Hence, there is an urgent need for innovative and effective alternative therapies against VL–HIV coinfection.

### Promising Role of Immunomodulatory Therapy

It has become increasingly clear that the host immune response is a critical factor determining VL treatment response and control, acting in synergy with anti-leishmanial drugs (37). This implies that in immunosuppressed individuals, targeting parasites alone with conventional anti-leishmanial drugs but without enhancing the immune response might simply not be sufficient. This interaction between drugs and the immune system was first suggested in animal models of VL, where the efficacy of pentavalent antimony (Sb^v^) was lower after T cell depletion (38). This was probably related to the decreased cellular uptake of Sb^V^ into interferon-gamma (IFNγ) activated macrophages, where it is normally converted intracellularly into its active trivalent form (Sb^III^) (4). While this finding should be extrapolated with caution, this mechanism may explain the observations that immunocompromised patients with VL failed to respond to antimonial drugs.

Immunotherapy is defined as the use of biological molecules or pharmacological compounds to modulate immune responses directly or in combination with drugs. A combination of immunomodulatory and direct anti-parasitic drugs could enhance the efficacy of chemotherapy and even prevent drug resistance (39). On top of its successful use in treating several non-infectious disorders (e.g., cancer, rheumatoid arthritis, etc.), the use of immune-based combination therapy is increasingly being explored in infectious diseases, such as tuberculosis (40) and leprosy (41). Despite several candidates being in the drug development pipeline, there are no immunotherapeutic agents or vaccines against VL currently registered for human use in routine clinical practice due to multiple reasons (e.g., high costs of clinical trials, limited and remote patient populations, ineffectiveness, safety concerns) (42). Experimental immune-based approaches are also being explored in the domain of HIV, where many have reached Phase I and some Phase II clinical trials but as of today have failed to provide enough immune restoration, potent effectiveness, sustainable benefits, delay of clinical progression, or good safety profiles (40, 43–46). However, VL–HIV-coinfected patients are often excluded or neglected in such studies, although both individual patients as well as public health approaches in general could benefit from these interventions.

Here, we first summarize current views on how host immunity against VL is affected during HIV coinfection, and then discuss the potential of current immunomodulatory therapies against VL in the context of concurrent HIV infection (both human studies and promising experimental approaches, excluding prophylactic studies). In particular, key targets and potential caveats are emphasized to guide future research on immunomodulatory therapies against VL and support the inclusion of HIV-coinfected patients in clinical research.

## Immunopathogenesis of VL–HIV Coinfection

**Figure 1:**
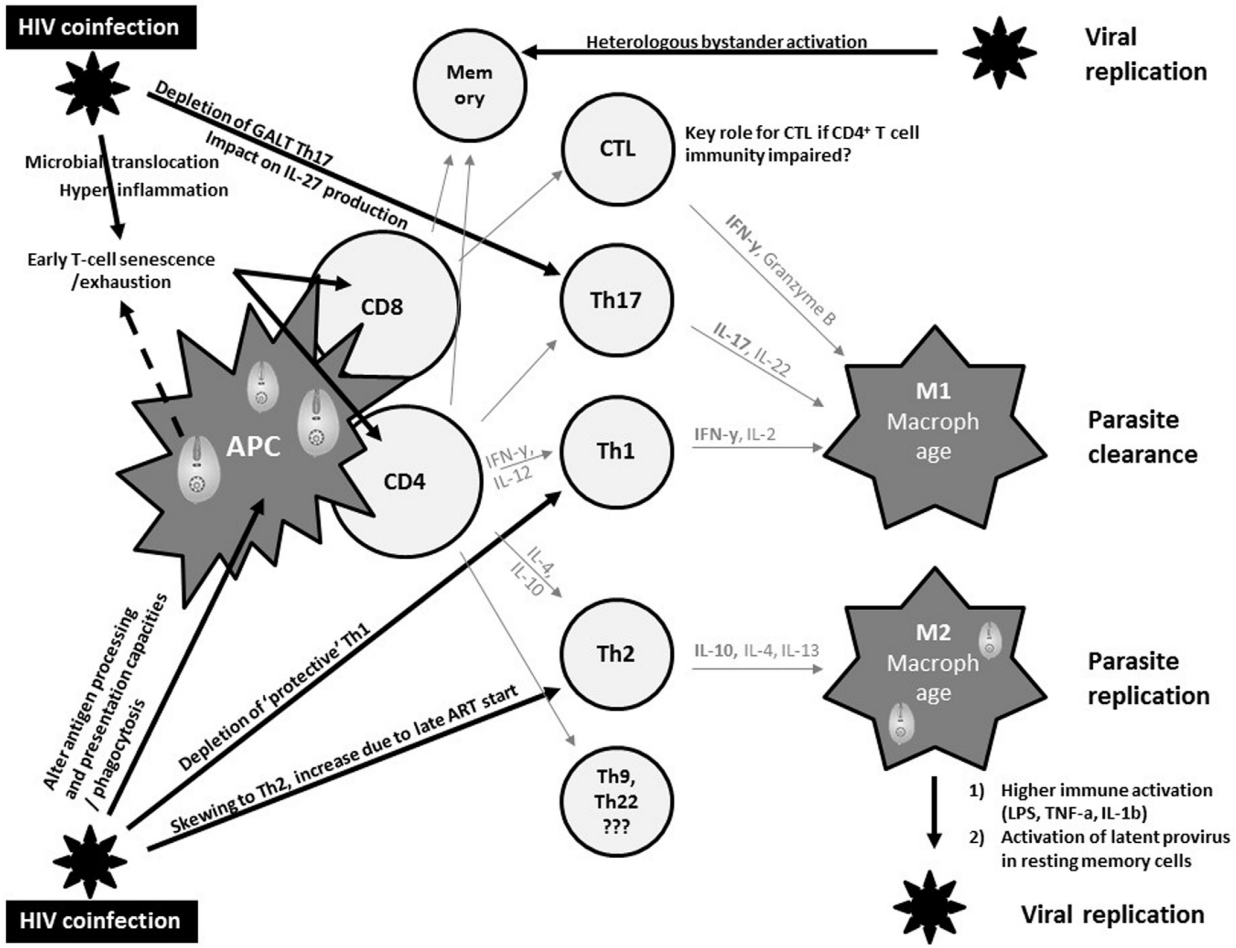
Current views on synergistic mechanisms in T cell immunity against visceral leishmaniasis (VL) due to human immunodeficiency virus (HIV) coinfection inciting persistent viral and parasite replication in VL–HIV-coinfected patients. APC, antigen-presenting cell; Th, T-helper; GALT, gut-associated lymphoid tissue; CTL, cytotoxic T cell; IL, interleukin; ART, antiretroviral therapy; IFN, interferon; LPS, lipopolysaccharide; TNF, tumor necrosis factor.

Macrophages represent an important common reservoir for HIV and *Leishmania* and serve as vehicles that disseminate both virus and parasite throughout the host. In addition, both pathogens may interact with each other to exacerbate immune suppression (Figure 1). In fact, both pathogens severely alter the antigen processing and presentation capacities of dendritic cells and macrophages, and synergistically escape immune surveillance using an array of strategies yet to be fully understood (47).

The control of VL in experimental models has been robustly associated with a strong T helper 1 (Th1) immune response, with large amounts of IL-2 and IFNγ (48) (Figure 1). In addition, a M2 polarization of macrophages has been associated with suppression of cell-mediated immunity, which confers susceptibility to intracellular infection. However, the immune mechanisms modulating VL in murine models or humans differ significantly. Human studies have shown a Th1/Th17 protective pattern with a somewhat different T cell functionality compared to experimental models, but lack comprehensive longitudinal data (49, 50). CD8^+^ T cells have also been shown to produce IFNγ that can contribute to VL control (51). The immunosuppressive effects of IL-10, and the regulatory role of other cytokines such as IL-27, have been implicated in the development of the different clinical pictures (50). Impaired neutrophil effector function has also been suggested to play a key role in the pathogenesis of VL (52). Partly due to the lack of good animal or *in vitro* models, it is currently unknown whether and how these protective and immunosuppressive patterns of VL are modulated by HIV and ART and how they define the pertinent clinical outcomes of VL–HIV patients.

Human immunodeficiency virus-1 causes a general profound impairment of cell-mediated immunity with low levels of CD4^+^ Th1 cells, the main protective cells in VL (Figure 1). HIV also skews the host immunity toward a Th2 response that only becomes affected at the later stages of the viral infection, potentially provoking parasite replication. Th17 cells are also associated with protection in VL, but are highly permissive to HIV infection. Their frequency is significantly and preferentially reduced in the gastrointestinal tract, even in patients with undetectable plasma viral load under ART (53). Depletion of Th17 cells from the gut-associated lymphoid tissue together with a series of immunopathological events occurring at the gastrointestinal tract mucosa leads to microbial translocation and consequently higher non-specific immune activation and hyper-inflammation (54). This microbial translocation has been postulated as one of the factors causing non-specific early T cell exhaustion and senescence (55), which may further weaken protective immunity toward VL. Likewise, VL was reported as an independent cause of increased non-specific immune activation, T cell senescence and the lack of immune recovery in virologically suppressed coinfected HIV patients (56, 57). In line with T cell exhaustion, chronic immune activation was recently associated with recurrent relapse of VL in HIV patients (58). Recent research in VL–HIV patients also suggested that weak antigen-specific functional responses or proliferation of T cells after *in vitro* stimulation was an important predictor of relapse (59). Despite the pivotal role of CD8^+^ T cells in viral and parasite clearance, their contribution in VL–HIV control and level of exhaustion remains unknown. Likewise, it is still unclear as to what impact *Leishmania* infection could have on the capacity of resting memory CD4^+^ T cells to act as a stable reservoir of latent HIV infection. What impact a spike in viral replication may have on anti-leishmanial immunity (e.g., by bystander activation of *Leishmania*-specific memory cells) also remains unknown (60, 61).

The consequences of infection by two immune-suppressive pathogens could, therefore, be a symbiotic and persistent incapacitation of the host’s immune system, favoring a state of immunological anergy, ultimately being fatal to the patient. A better understanding of the immune response against *Leishmania* infection in HIV-coinfected patients is crucial to establish a rational approach for immunomodulatory therapy.

## Status of Immunotherapeutic Interventions in Human VL and Their Application in HIV Patients

Due to the lack of a protective role of anti-*Leishmania* antibodies in early studies, passive immunization was not further explored, while active immunization with immunomodulators and vaccine therapy was investigated (62). Early studies by Murray et al. (38, 63, 64) showed the therapeutic utility of interleukin-2 (IL-2), IL-12, interferon-gamma (IFNγ), and granulocyte–monocyte colony-stimulating factor (GM-CSF) in murine VL models. Although the Th1/Th2 dichotomy of immunity to VL is not fully upheld in humans, clinical immunotherapeutic studies on VL patients have been skewed toward Th1-associated cytokine-adjuvant therapy and are discussed below (see Table 2). For VL–HIV coinfection, only five published case reports using recombinant IFNγ, IL-2, and GM-CSF combined chemotherapy were found in literature (see Table 2).

**Table 2:** Published clinical reports on the use of immuno(chemo)therapy against visceral leishmaniasis (VL) and VL–human immunodeficiency virus (HIV).

Reference	Country; year; design	Patient characteristics	Chemo agent	Immuno agent	Outcome (EOT)	Comments
**VL mono-infection**						

(65)	Brazil; 1990; case series	(1) SSG-unresponsive VL (*n* = 8); <18 years (8/8); Mean age: 6.5 years	SSG 20 mg/kg	IFNγ (100–400 µg/m^2^ for 10–40 days)	6/8 cured EOT (75%) No relapse during study period	Higher cure rates in both groups compared to historical controls Tolerability acceptable (fever)
		(2) Severely ill primary VL (*n* = 9) <18 years (8/9) Mean age: 9.8 years	SSG 20 mg/kg	IFNγ (100–400 µg/m^2^ for 10–40 days)	8/9 cured EOT (89%) No relapse during study period	

(66)	Brazil, 1993; case series	(1) Primary VL (*n* = 8) Predominantly children Median age: 5 years (2) SSG-unresponsive refractory VL (*n* = 14) Median age: 4 years	SSG 20 mg/kg SSG 20 mg/kg	IFNγ (100–400 µg/m^2^ for 10–40 days) IFNγ (100–400 µg/m^2^ for 10–40 days)	8/8 cured EOTCure 12 M: 8/8 (100%) 1/8 relapsed 12/14 cured	Both groups: more severe cases than in 1990
					Cure 12 M: 9/14 (64%) 6/12 relapsed	

(67)	Kenya; 1993; randomized controlled trial (RCT)	(1) Primary VL (*n* = 10) <18 years: 7/10 (2) Primary VL (*n* = 14) <18 years: 11/14	SSG 20 mg/kg SSG 20 mg/kg	IFNγ (100 µg/m^2^ every –2–30 days) /	24/24 cured EOT Week 1:50% cured Week 2:75% cured Week 4:100% cured Week 1:22% cured Week 2:58% cured Week 4:88% cured	Control group included no relapse cases a non-significant accelerated response with SSG + IFNy

(68)	Brazil; 1994; RCT	(1) 10 neutropenic primary VL (2) 10 neutropenic primary VL	SSG 10–20 mg/kg for 10 days SSG 10–20 mg/kg for 10 days	Granulocyte–monocyte colony-stimulating factor (GM-CSF) (5 mg/kg for 10 days) Placebo	Cure M3: 100% Cure M3: 100%	Study focused on hematological evaluation and secondary infections Secondary infections occurred in 3 GM-CSF and in 8 placebo recipients

(69)	India; 1995; RCT	(1)Primary VL (*n* = 16)Mean age 21 years (range 6–52)	SSG 20 mg/kg for 20–30 days	IFNγ (100 µg/m^2^)	Cure D10: 10/15 (63%) Cure D20: 14/15 (93%) Cure D30: 15/15 (100%) Cure M6: 13/15 (87%)	D10 and D20 difference statistically significant No relapse up to M24
		(2) Primary VL (*n* = 15)Mean age 27 years (range 5–58)	SSG 20 mg/kg for 20–30 days	/	Cure D10: 1/15 (7%) Cure D20: 6/15 (40%) Cure D30: 11/15 (73%) Cure M6: 9/15 (60%)	Treatment was discontinued early in the 14 IFNγ treated responders after D20

(70)	India, 1997	(1) Primary VL (*n* = 52) Mean age 20 years; 60% male (2) Primary VL (*n* = 52) Mean age 18 years; 58% male (3) Primary VL (*n* = 52) Mean age 20 years; 69% male	SSG 20 mg/kg for 30 days SSG 20 mg/kg for 30 days SSG 20 mg/kg for 30 days	IFNγ (100 µg/m^2^ for 30 days) IFNγ (100 µg/m^2^ for 15 days) /	Cure (EOT): 25/47 Relapse: 1 6 M cure: 24/49 (49%) Cure (EOT): 22/50 Relapse: 1 6M: 21/50 (42%) Cure (EOT): 20/48 Relapse: 2 6 M cure: 18/50 (36%)	High failure rate with standard therapy (SSG-resistance?) Differences not statistically significant

(71)	USA, 2012, Phase I RCT	(1) Healthy volunteers (*n* = 12) (2) Healthy volunteers (*n* = 12) (3) Healthy volunteers (*n* = 12)	/ / /	Leish F3 (20 μg) + GLA-SE (5 μg) Leish F3 (20 μg) + GLA-SE (2 μg) Leish F3 (20 μg)	Safe and immunogenic D84: 10/10 Safe and immunogenic D84: 8/8 Safe and immunogenic D84: 9/9	Subunit vaccine: single recombinant fusion protein of 2 preserved proteins

(72)	UK, 2016, Phase I trial	(1) Healthy volunteers (*n* = 20) *n* = 5 low dose*n* = 15 high dose	/	ChAd63-KH (1 × 10^10^ vp or 7.5 × 10^10^ vp)	Safe and immunogenic D90: 20/20	Adenovirus vector encoding 2 Leishmania proteins Dose escalation study

**HIV and VL coinfection**						

(73)	CASE REPORT; 1990	Full-blown acquired immunodeficiency syndrome (AIDS) patient with recurrent VL 19-year-old Algerian male	Meglumine antimoniate (dose unknown) Pentamidine (2 mg/kg iv 3 times/week, 1 week/month)	IFNγ (175 μg/day iv or sc for 21 days) IFNγ (175 μg/day sc 3 times/week, 1 week/month)	1 relapse treated Resistance to antimoniate 3 relapses treated Cure 6M: Only two mild relapses with minimal adverse events (AEs)	

(74)	CASE REPORT; 1993	Three full-blown AIDS patients	Meglumine antimoniate (dose unknown)	IFNγ (dose unknown)	Clinical improvement Reduction in parasite burden	

(75)	CASE REPORT; 1994	Full-blown AIDS patient with Kaposi syndrome (KS) 40-year-old German male	SSG (dose unknown)	IFNγ (dose unknown)	Aggravated KS	

(76)	CASE REPORT; 2004	Primary VL 37-year-old Italian male CD4 < 50 mcl On ART	Amphotericin B (4 mg/kg for 5 days + 5 non-consequent days)	GM-CSF(150 mcg/twice a week for 12 weeks)	Dramatic Clinical improvement No AEs	

(77)	CASE REPORT; 2007	Unresponsive VL 36-year-old Italian woman CD4: 98 cells/μl On ART	Amphotericin B(between every cycle)	IL-2 (twice/day for 5 days—7 cycles every 4–8 weeks) (cycle 1–4: 3MIU; cycle 5–7: 6MIU)	No benefit Increase in *Leishmania* DNA	

*IFN, interferon; SSG, sodium stibogluconate; VL, visceral leishmaniasis; EOT, end of treatment*.

### Interferon-γ

There has been limited success in small-scale clinical trials with combined therapy of IFNγ and Sb^V^ for treating VL. This combination therapy displayed stronger parasitological and clinical cure rates in VL patients (mainly children) from Brazil, Kenya and India compared with the drug alone, but these studies had several limitations (see Table 2 for details). In a subsequent larger randomized controlled trial (RCT) in India, these improved treatment outcomes could not be confirmed (70). Importantly, treatment response in this particular study was generally poor as drug resistance was emerging in that region.

There are a few case reports, mostly from the pre-ART era, providing information on whether IFNγ can be safely administered in VL–HIV patients (see Table 2), which is of relevance since IFNγ also has a vital but ambiguous role in the pathogenesis of HIV (78). IFNγ appeared to be fairly well tolerated but showed inconclusive results (73, 74, 79). In one old case report of a patient with VL–HIV coinfection, acceleration of Kaposi’s sarcoma has been reported (75). The therapeutic potential of IFNγ to treat HIV coinfections was supported by two Phase II trials, evaluating adjunctive IFNγ to improve treatment response to antifungals in HIV patients with cryptococcal meningitis (80, 81). However, in the early 1990s, a multicenter clinical trial of SSG plus IFNγ for VL in HIV-coinfected patients in Spain was suspended following an interim analysis indicating that there was an excess of severe secondary effects and no benefit over drug alone (79). The findings itself have never been published but suggested a limited value of IFNγ therapy for VL–HIV coinfection.

### Granulocyte–Macrophage Colony-Stimulating Factor

Granulocyte–monocyte colony-stimulating factor can inhibit the intracellular replication of protozoa such as *Leishmania*. The justification to explore GM-CSF as immunotherapeutic agent stems from documented effects, such as monocyte mobilization, macrophage activation, the production of pro-inflammatory cytokines, and amelioration of neutropenia (63). GM-CSF combined with Sb^V^ was successfully explored in 20 neutropenic VL patients in Brazil. All responded well to VL treatment, neutropenia rapidly improved and secondary infections decreased (68) (Table 2). The authors did, however, not include a control arm, making it unclear whether the effect of GM-CSF, if any, could be due to the reversal of neutropenia (and might hence not apply in those without neutropenia) or whether other mechanisms were involved. On the other hand, *in vitro* studies have recently suggested that GM-CSF could contradictory promote *Leishmania* growth by inducing monocyte proliferation and induction of intracellular dNTP production (82), but whether this would also occur in humans remains unknown.

In terms of safety, several older clinical trials of GM-CSF administration in HIV patients indicated that it might accelerate HIV replication (83). By contrast, more recent RCTs have demonstrated benefits of using GM-CSF in virologically suppressed patients as an adjunct to conventional ART or therapeutic HIV vaccination (83, 84). This would argue against using GM-CSF in pre-ART patients, but might suggest it to be safe in those stable on ART. With regard to coinfections, some case reports were published on successful GM-CSF therapy of resistant-to-standard-therapy mycobacterial infection and pulmonary aspergillosis in HIV patients (85, 86). There is a single successful case report on immunotherapy targeting primary VL in an Italian AIDS patient, whereby human GM-CSF was combined with liposomal amphotericin B (Table 2) (76). Presently the evidence for beneficial effects of GM-CSF on HIV disease is limited, but GM-GSF adjuvant therapy could provide a potential value for treatment of neutropenic VL in stable ART patients.

### Interleukin-2

Interleukin-2 induces clonal expansion of specific T cells; promotes natural killer and CD8^+^ T cell cytotoxicity, cytokine secretion by Th1, Th2, and Th17 cells; and modulates programmed cell death (42). Hence, IL-2 is necessary for the protection against *Leishmania* in immunodeficient mice, in which IL-2 restores the activity of Sb^V^ (38, 87). The impairment in IL-2 production is also one of the first functional defects described in untreated HIV-positive patients and its administration to boost the quantitative and/or qualitative CD4^+^ T cell restoration in HIV-infected patients has been evaluated in Phase I, II and III trials (42). These early results provided evidence that IL-2 therapy combined with existing cART has the potential to enhance quantitative and qualitative immune restoration, without triggering HIV replication, even when ART alone had failed to do so. However, restoring CD4^+^ T cell counts with IL-2 failed to show long-term clinical benefits in two large Phase III clinical trials, ESPRIT and SILCAAT (88). IL-2 recipients in the STALWART trial even experienced more opportunistic infections, death or grade 4 adverse events during IL-2 administration, than those not receiving IL-2 (89).

To date, no clinical trial for rIL-2 administration in VL patients has been reported. There has been one case report on the use of rIL-2 in a VL–HIV-coinfected patient failing to respond to anti-leishmanial and HIV treatment with low CD4 counts and incomplete HIV suppression despite ART use (77). This report indicated no benefit. Importantly, increased *Leishmania* parasitemia was observed at each rIL-2 cycle, which might have favored the progression of HIV infection and possibly explains the reported progressive decline in CD4 T cell count (77). In a BALB/c mouse model, IL-2 seemed to have a short protective effect against VL only at the priming phase, without any lasting benefit (90). Such a phase-specific effect could explain the lack of long-term clinical benefits. In general, the small therapeutic window, critical dosage with potential high toxicity and challenging treatment conditions suggest IL-2 is an unlikely candidate for boosting immunity in VL–HIV-coinfected patients.

### Therapeutic Vaccines

Historically, leishmanization (inoculation with live parasites) was shown to have benefit for protection against re-infection with cutaneous leishmaniasis (CL) and this evidence has driven the search for an effective vaccine against VL (91). Besides prophylactic vaccine development, various approaches employing therapeutic vaccines have been tested experimentally and clinically; and currently resulted in three licensed vaccines for canine VL but none for human VL (92). Therapeutic immunization with a first generation vaccine of aluminum hydroxide precipitated autoclaved *L. major* (Alum-ALM)+Bacille Calmette–Guérin (BCG) was found clinically effective in CL, mucocutaneous leishmaniasis and persistent post-kala-azar dermal leishmaniasis (PKDL) cases, with studies progressing to Phase III clinical trials (93–99), but application to VL has not been reported (62). Similarly, LeishF1/F2 vaccine (alternatively called Leish-111f), a promising second-generation (i.e., recombinant protein) vaccine for CL, showed insufficient protection against VL in dogs (100). A modified version of these second generation vaccines, called LeishF3, which accommodated changes to enhance its efficacy against VL has been shown to be safe and immunogenic in a Phase I trial in healthy human volunteers, but therapeutic trials in patients have not been reported (Table 2) (71). A third-generation (i.e., DNA-based) adenovirus vaccine (ChAd63-KH) was designed to induce *Leishmania*-specific CD8^+^ T cells and aimed at therapeutic use in VL/PKDL patients. It was shown to be safe and immunogenic in healthy volunteers (72) and is currently in Phase II trial in persistent PKDL patients in Sudan.

A careful risk–benefit assessment needs to be made when considering therapeutic vaccination against VL in HIV patients, with depressed immunity. Safety concerns surely exist, but should not be overstated and should not impede evaluation of therapeutic VL vaccination studies in virally suppressed HIV patients as potential benefits can outweigh existing theoretical risks. In essence, these patients have a higher risk of developing VL and are most in need of an enhanced immune response upon VL development. Post-marketing trends suggest that routinely used inactivated (non-VL) vaccines have similar safety profiles among HIV-uninfected and HIV-infected persons on stable ART (101). Although data are still limited, HIV-infected individuals who are on ART with well-controlled HIV RNA levels and CD4^+^ T cell counts of >200 cells/μL (or ≥15%) may even receive indicated live-virus vaccines (101). In addition, modern post cART era studies did not indicate that vaccines are important triggers of HIV replication or disease progression (102). With regard to efficacy, a highly immunogenic vaccine will be needed, as well as detailed studies to define the optimal timing and dosing for vaccination among those with advanced disease. Despite the concerns of depressed immunity and sparse efficacy data for other types of vaccines, studies have clearly demonstrated the protective benefit of influenza and *Streptococcus pneumoniae* vaccinations even among advanced HIV patients. In summary, these data merit a concurrent evaluation of therapeutic VL vaccines in coinfected patients who are virologically suppressed at the time of VL presentation.

## Promising Pipeline Immunomodulatory Molecules/Interventions

**Figure 2:**
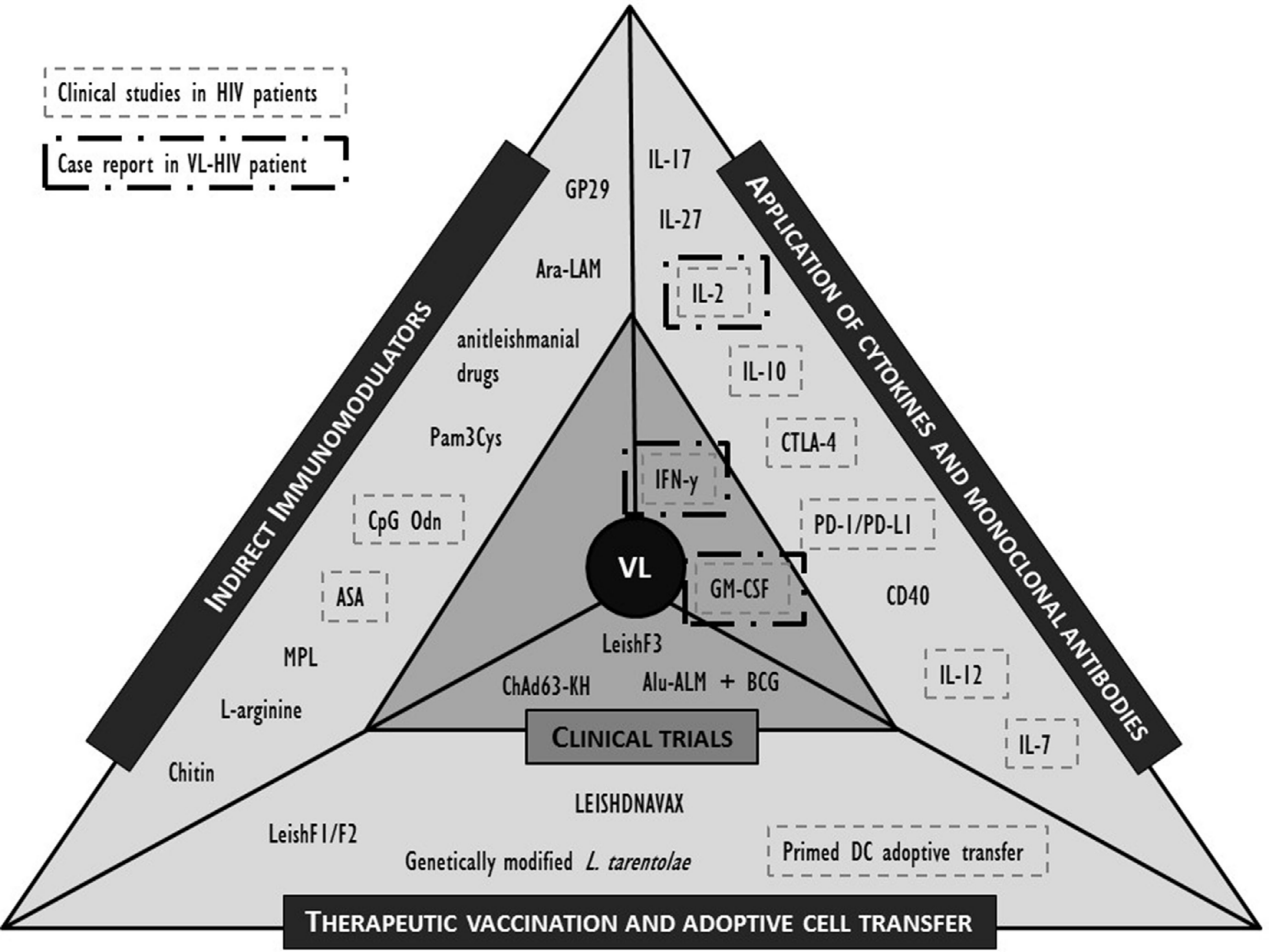
Overview of described clinical and preclinical immunomodulatory interventions in human visceral leishmaniasis (VL) and their application in (VL)-human immunodeficiency virus (HIV) (co)infection. IL, interleukin; IFN, interferon; PD-(L)1, programmed cell death-(ligand)1; GM-CSF, granulocyte–macrophage colony-stimulating factor; CTLA, cytotoxic T lymphocyte-associated molecule; CD, cluster of differentiation; BCG, Bacillus Calmette–Guérin; Alu-ALM, aluminum hydroxide precipitated autoclaved L. major; DC, dendritic cell; GP, Glycoprotein; Ara-LAM, arabinosylated lipoarabinomannan; Pam3Cys, synthetic bacterial lipopeptide; CpG Odn, CpG oligodeoxynucleotides; ASA, acetyl salicylic acid; MPL, monophosphoryl lipid.

While both the pharmacokinetics and pharmacodynamics of a drug, but also the nature of drug–immune interactions in animals and humans may differ considerably, animal models may still provide new clues to potential approaches. Here, we selected the most promising molecules or interventions for their potential in an immunosuppressive environment of the coinfected individual and refer to recent review papers for a more extensive list (39, 44, 45, 92, 103). The formats discussed below are limited to active immunotherapy attempts including non-antigen-specific strategies such as cytokines that stimulate immunity or suppress the viral replication; antibodies that block negative regulatory pathways; and indirect immunomodulation (Figure 2). Antigen-specific strategies such as therapeutic vaccination and adoptive strategies such as cell therapy are also briefly discussed. Whether the molecules listed below could serve as putative targets for human immunotherapy remains to be demonstrated.

### Non-Antigen-Specific Strategies

The above listed clinical trials with cytokine-adjuvant chemotherapy were based on limited data from experimental models of VL conducted in the 1990s. Our knowledge of immune mechanisms has substantially expanded since then. For instance, IL-12, a pluripotent cytokine that plays a central role in the initiation/maintenance of Th1 responses and potentiates T cell IFNγ production, was shown to have similar effects as IFNγ in both CL and VL when injected in mice (64, 104) as well as dogs (105) and human PBMC from treated Sudanese VL patients (106). Likewise, IL-12 preconditioning of monkeys during acute SIV infection markedly delayed disease progression (107). While rhIL-12 administration was well tolerated and safe, no evidence of improvement in HIV antigen-specific immune response could be observed in a Phase I RCT (108). While this suggests that IL-12 therapy is unlikely to provide major benefits in the chronic phase of an HIV infection, it might still be valuable in the context of opportunistic infections that are best met with Th1-like effector immune responses. In line with this, rIL-12-adjuvant chemotherapy was successfully evaluated for patients with Kaposi’s sarcoma (109). In addition, it has been tested as part of a combination therapy for cryptosporidiosis in two AIDS patients that demonstrated signs of a brisk immune response and consequently symptomatic improvement, but with severe side effects that outweighed the clinical benefits (110). Data on the role of IL-12 as an immunotherapeutic agent or vaccine adjuvant for HIV coinfections could be promising and merits further research, although potential broad side effects due to its pluripotent role should be limited (e.g., tissue-targeted delivery, well-timed short boosting approach, etc.). Unfortunately, the incorporation of IL-12 into larger vaccine trials has lagged, largely due to the early setback in a renal carcinoma Phase II trial. However, the mechanisms underlying the severe acute toxicities that led to two deaths and 12 hospitalizations have been ascribed to an inappropriate dose and administration schedule (111).

Like IL-12, many chemokines or cytokines contributing to protection/pathogenesis of VL are regulated during HIV coinfection. For instance, Th17 cells are highly depleted from the gut in HIV-infected patients. Recent work in humans has, however, demonstrated the importance of IL-17 and IL-22 in protection against VL progression from asymptomatic infection to disease (49). In addition, elevated serum IL-27 concentrations were linked to severity of VL. IL-27 seems to regulate the Th1/Th17 profiles in a *L. infantum* mouse model of VL by suppressing the IL-17-induced neutrophil response (112). The IL-27–Th17–IL-17 axis, thus, seems to be strongly involved in resistance against VL and merits further therapeutic exploration, especially in HIV-coinfected patients with a Th-17-depleted immune response.

Despite the central role of IL-7 cytokine therapy in HIV patients in the past, this molecule has not been evaluated in VL–HIV-coinfected patients and remains under-investigated in experimental models of VL (113). IL-7, like IL-2, has a critical role in peripheral T cell homeostasis. IL-7 has, however, a more pleiotropic role and was shown to drive CD4^+^ T cell restoration in HIV patients, even when HIV replication is controlled. It is also able to promote Th1 responses, enhance memory T cell expansion (on top of naive T cell response), and increase CD8^+^ T cell counts and cytotoxicity in HIV patients (42). Moreover, damage to hepatocytes during full-blown VL may impair IL-7 production, as IL-7 is also produced by liver cells in response to inflammation (114). Recombinant IL-7 administration thus has the potential to safeguard the long-term survival of effector CD4^+^ T cells in response to persisting parasites in a VL–HIV coinfection. However, in the ERAMUNE 01 RCT, rIL-7 and dual ART intensification induced an amplification of the HIV reservoir in well-controlled HIV patients (115). The authors reasoned that this was the result of the expansion of central memory CD4^+^ T cells, carrying HIV DNA, thus limiting this IL-7 based strategy. In the context of VL–HIV coinfection, this strategy should only be considered if a pronounced clinical benefit to VL treatment outweighs its potential negative effects.

Blocking the action of immune-suppressive factors could prove more efficient as it might allow restoration of protective immunity in a more controlled manner. IL-10 correlates very well with the parasite load during VL infection. Moreover, in animals, IL-10 blockade (by means of anti-IL-10R or anti-IL-10 monoclonal antibody) has been proven successful in lowering parasite burden when combined with conventional treatment in multiple studies in mice (116, 117). These effects were confirmed in cultures of splenocytes or PBMCs from Indian and Sudanese VL patients (106, 118). However, in immunodeficient mice treated with anti-IL-10R monoclonal antibody, Murray et al. were not only able to show an acceleration of Sb^V^-associated killing, but also reported a >10-fold Sb^V^ dose-sparing effect (119). Despite the clinical and experimental data suggesting IL-10 as a key target in the immunopathogenesis of VL, a clinical trial using a monoclonal antibody against IL-10 failed to start following the decision of the company to stop its production (NCT01437020, clinicaltrials.gov).

Increased serum IL-10 concentrations are also observed in HIV-infected patients with disease progression, in contrast to non-progressing patients where levels were stable (120). In addition, ART has a clear downregulating effect on IL-10. On the other hand, increasing evidence suggests that IL-10 impacts many aspects of HIV pathogenesis, including the regulation of HIV-specific CD4^+^ and CD8^+^ T cell functions, as well as modulation of HIV replication in PBMC subsets. Genetic polymorphisms in the IL-10 gene promoter that lead to decreased IL-10 expression have been associated with more rapid disease progression in late stages of HIV infection, suggesting that the anti-inflammatory effects of IL-10 may be solely protective in the setting of chronic immune activation and blocking IL-10 function would only make sense in an acute setting (121). When considering VL–HIV coinfection, these data would advocate the blocking of excessive IL-10 levels during the acute stage of VL in HIV patients (in particular pre-ART patients) to allow a beneficial acute response which should however be time limited to retain the beneficial role of IL-10 in controlling side damage of chronic HIV and parasitic infections. To reduce the unwanted side effects due to blockage of normal, and beneficial, biological activities, novel IL-10 signaling inhibitors with for instance shorter half-lives are first needed (122).

The concept of immune exhaustion and senescence as a stepwise and progressive loss of T cell function and proliferative potential, respectively, and evolving to complete T cell unresponsiveness has been robustly discussed in the context of HIV infection (123). The driving force is believed to be chronic antigen exposure and consequently extensive non-specific immune activation. Increased immune activation in patients on long-term suppressive cART has been associated with increased mortality, the occurrence of non-AIDS-defining conditions, and a poorer recovery in CD4^+^ T cell count (124, 125). Similarly, increased levels of programmed death-ligand 1 (PD-L1) expression on monocytes, B cells, and T cells from untreated HIV patients correlated directly with plasma viral load and inversely with CD4^+^ T cell count (126). This mechanism could partly explain the disappointing long-term effects of IL-2 therapy in HIV patients, as IL-2 was recently shown to upregulate the PD1–PD-L1/L2 pathway (127). While the causative factors of immune exhaustion or senescence are not completely understood, chronic immune activation, residual HIV-replication, and coinfections are likely main drivers of this process. Recent studies have also focused on the role of this process in the context of VL and other parasitic infections, showing an accelerated T cell senescence during VL infection (128). Likewise, a parasite-induced T cell anergy has been proposed (128). Hence, a modulatory approach to reverse this process or temporarily breaking the regulatory feedback loop using antibody therapies targeting PD-1, CTLA-4 or its ligands could prove efficient in coinfected individuals with a potential double-driven T cell unresponsiveness. Such an approach to reverse the reported T cell unresponsiveness has proved very effective in experimental VL (57, 129–131). In SIV-infected rhesus macaques, anti-PD-1 (in the absence of ART) was shown to enhance virus-specific CD8^+^ T cell activity, to reduce viral load, and to prolong survival (46). Similarly, anti-PD-L1 antibody therapy showed promising in a recent Phase I RCT on 6 ART patients, arguing in favor of its potential use in virologically suppressed VL–HIV patients (132). Recently, the major HIV cell reservoir was shown to be composed of PD-1^+^ CD4^+^ memory T cells, suggesting an additional positive effect of anti-PD-1 therapy to combat the concomitant HIV infection (133).

### Antigen-Specific and Adoptive Strategies

There are multiple studies in which diverse antigens and adjuvants showed promising results as immunoprophylactic or therapeutic tools in animal models of VL, recently summarized in a review by Jain and Jain (92). Apart from the current clinically explored strategies and the safety/efficacy concerns in HIV patients (see above), a promising approach would be to vaccinate with a non-pathogenic *L. tarentolae* strain, genetically modified to improve its immunogenic potential as a live vaccine (134). Likewise, a novel third generation T cell epitope-enriched DNA vaccine (LEISHDNAVAX) showed significant efficacy when co-administered with a single dose of AmBisome in *L*. *donovani*-infected mice (135). The vaccine is based on minimalistic immunogenically defined gene expression vectors encoding five conserved antigens developed for efficient induction of Th1 immune responses. This candidate vaccine has yet to enter clinical Phase I trials.

Another cutting-edge approach to induce antigen-specific T cell immunity is dendritic cell-based immunotherapy (103, 136). While macrophages are one destination of *Leishmania* parasites in the human host, dendritic cells can also harbor parasites, but in addition present antigen and regulate immune mechanism governing control or progression of infection. Adoptive transfer of dendritic cells primed with different kinds of *Leishmania* antigens has been shown very effective in murine VL, improving both cellular and humoral immunity (136). Compared to the modest efficacy of immune therapy and therapeutic vaccines against HIV infection, *ex vivo* generated dendritic cell therapeutic vaccines aimed at inducing effective HIV-specific immune responses have yielded the best results in this field (137). The outcomes of monocyte-derived dendritic cell-based therapeutic vaccines still needs optimization as functional cure was not reached and most patients needed to restart ART, but this method could provide a strong immunogenic window for concomitant VL-targeted therapy of coinfected individuals. Due to high costs and required state-of-the-art equipment, adoptive cell transfer therapy may prove difficult to implement in low-resource settings of disease endemic countries.

### Indirect Strategies

An alternative approach is to indirectly stimulate host immunity to optimize protection against infection. Such indirect immunomodulators can be obtained by many different types of substances, including natural products that have immunomodulatory activity. Such immunomodulators, however, carry the risk of inducing excessive immunopathology and side effects. Many compounds have been evaluated in VL animal studies over the years, including CpG oligodeoxynucleotides, acetyl salicylic acid and l-arginine (103). Most of these molecules increase T cell activation through enhanced antigen presentation by costimulation-based therapy or acting on toll-like receptors (TLRs) (e.g., TLR4/GP29 or MPL; TLR2/Ara-LAM, or Pam3Cys). This could be particularly beneficial in HIV-coinfected patients, as TLR-agonists such as TLR7 or TLR9 agonists have shown reduction of viral DNA or the viral reservoir and enhancement of HIV-specific CD8^+^ T cell immunity in experimental and human HIV (138, 139). Whether such a multi-TLR targeting approach would benefit human VL–HIV patients remains unclear and merits further research.

In a similar manner, it has been suggested that TLR4 and TLR9, two TLRs contributing to the immune response against *Leishmania* infection, play a role in the anti-leishmanial mechanism of miltefosine (140). An alternative strategy could, thus, be to concurrently capitalize on the indirect immunological effects of the combined anti-leishmanial drug in a immuno-chemotherapeutic approach. The relevance and impact of these immunomodulatory actions of current anti-leishmanials in HIV-coinfected VL patients remains to be determined. Besides a direct mechanism of action, anti-leishmanials can increase nitric oxide and reactive oxygen species production due to activation of infected macrophages, leading to elimination of the parasite. This indirect activation of macrophages has been shown for amphotericin B (141), miltefosine (142), antimonials (143), and paromomycin (144). Induction of macrophage-derived cytokine release promoting a Th1 response (IL-2, IL-12, IFNγ) has been noted for all conventional anti-leishmanials such as amphotericin B (141, 145), miltefosine (142, 145), paromomycin (145), and SSG (143, 145), even though contradictory results have been reported, e.g., for miltefosine (146). Related to this, miltefosine restored IFNγ responsiveness in *Leishmania-*infected macrophages (142). Another immunostimulatory property contributing to anti-leishmanial activity is a drug-induced increase in macrophage membrane fluidity, ameliorating defects in antigen presentation and enhancing T cell stimulation. This has been shown after exposure of infected host cells to higher concentrations of miltefosine, paromomycin, and SSG (145). For both antimonials (147) and miltefosine (148), it has been shown that they increase the phagocytic capacity of monocytes and macrophages. There are currently no data available whether all these effects are clinically relevant in terms of short-term treatment response, relapse, final cure, and the risk of development of PKDL. Despite the current lack of data on clinical relevance, these background effects should be taken into consideration in future combined immuno-chemotherapeutic strategies to incite an effective synergistic effect. The general lack of response to anti-leishmanial treatment in HIV-coinfected patients and the relevance of concomitant cART for the efficacy of current anti-leishmanials possibly indicate that these indirect effects are not negligible for a therapeutic response.

## Perspectives

Despite the growing research in immunotherapy against VL (partly reviewed above), no immunotherapeutic approach has yet been licensed for use in human VL. HIV-coinfected patient groups, in particular, are often excluded from the above described clinical intervention studies due to the presumed hazards and challenging logistics. Although a vulnerable population, we would argue that VL–HIV patients should be considered as a relevant target group for an immunomodulatory approach against VL due to an intensified defect in T cell immunity, dependence of current anti-leishmanial drugs on the latter, inadequate treatment outcomes, and higher chronicity of the parasitic infection with frequent relapse. In addition, HIV-targeted immunomodulatory approaches, despite their drawbacks to achieve long-term functional cure in HIV patients, might find a temporarily window of opportunity in opportunistic coinfections such as VL, where cART alone is not able to restore protective immunity. The challenge, however, of immunomodulatory therapy in VL–HIV-coinfected patients is boosting effective VL-specific T cell responses while avoiding activation of latent provirus and inappropriate immune activation (in virologically suppressed ART patients) or HIV recrudescence and increased HIV-susceptibility of target cells (in unstable HIV/AIDS patients). Clinical trials are a necessity to study treatment effects, due to the lack of good animal or *in vitro* models mimicking VL–HIV coinfection.

In Figure 2, we summarized the discussed interventions against VL and highlighted those that have also been clinically evaluated in the context of HIV. Evidence is lacking to prioritize a target molecule, but attempts at immunotherapy in VL–HIV patients should best be performed in ART patients with a recovered immune system. Appropriate adjuvants can be included to enhance the efficacy of the response, but caution should be taken to avoid excessive and broad immune activation. The following perspectives are best taken into consideration when designing or evaluating an immunomodulatory approach in VL–HIV-coinfected patients.

### Combination Strategies

As current anti-leishmanial drugs are highly dependent on host immunity, it is recommended to potentiate chemotherapeutic agents with various immunomodulators in HIV-coinfected patients. While the increment in immunocompetent patients could be potentially low, HIV-coinfected patients are probably in more need of a boost in effective T cell immunity against VL to decrease the high mortality and treatment failure rates typically observed in coinfected patients.

The current clinically explored techniques of single cytokine-adjuvant therapy in VL have the inherent danger of a very pluripotent effect in HIV-coinfected patients, due to the intricacies of cytokine networks, and may unpredictably impact the delicate balance between beneficial VL-specific responses and deleterious immune activation. Future therapeutic use of broad immunomodulators will most likely lead to unwanted side effects in coinfected patients until a system-level understanding of their mode of action is available and thus a more selective and well-timed approach can be performed (149). However, they could potentially prove valuable as a well-timed adjuvant in a more targeted immunomodulatory approach.

The other clinically explored strategy in VL is therapeutic vaccination. However, as T cell senescence and exhaustion could have occurred by persistent HIV replication, further stimulating effector-memory T cells could be futile or even harmful in VL–HIV patients. Perhaps a concurrent strategy to reverse this T cell exhaustion (e.g., anti-PD-1 therapy) could increase vaccine efficacy. It remains to be seen whether VL–based therapeutic vaccines deployed in HIV-coinfected patients are safe and whether a strong enough response can be induced against VL. In severely CD4^+^ depleted patients in particular, a concurrent need may be to first encourage immune reconstitution before vaccination. Combination strategies of diverse immunomodulators and drugs will, thus, be crucial in these patients to reach an effective treatment, perhaps with a more individualized approach.

### Stratification

Among patients with tuberculous meningitis, different inflammatory patterns governed by host genetics are recognized, converging on dysregulated levels of TNF. At one end of the extreme, a hyper inflammatory phenotype was shown to benefit from steroid administration; at the other end, where inflammation is inadequate, other immunomodulatory interventions would be required (150). In a similar manner, subgroup analyses in HIV-associated cryptococcal meningitis suggested that the greatest benefit of a short-course IFNγ adjuvant therapy was gained among patients with a lack of Cryptococcus-specific IFNγ/TNF CD4^+^ T cell responses (151). In most settings, VL–HIV-coinfected individuals will also be (severely) malnourished upon VL diagnosis, and micro- and macro-nutrient deficiency can have profound immunological effects. These alterations could critically affect the efficacy of any immunomodulatory interventions, yet may also provide opportunities for complementary interventions. We, therefore, argue that there is a need to assess immune risk profiles based on functional T cell assays, RNA signatures, and other parameters that identify patients that are more likely to benefit from immune adjuvant therapy, across the heterogeneous group of VL–HIV patients.

### Timing

Visceral leishmaniasis–HIV coinfection is a dynamic process with diverse stages of infection and regardless of choice of immunomodulatory intervention, timing will be critical to success. For instance, high IL-17 levels appeared protective for early VL progression, but its role is still debatable in chronic infection. The optimal timing of immunotherapy among HIV-coinfected adults in regard to HIV stage and receipt of antiretroviral therapy also remain important unanswered questions. Most benefit is probably to be gained in early stages of HIV infection as well as in under-therapy suppressed patients, who are able to effectively respond to immunomodulators. Therefore, we would argue for a primary evaluation of novel approaches in stable ART patients that have a somewhat reconstituted CD4^+^ T cell immunity and suppressed viral load, including frequent monitoring of blips in viral load and CD4^+^ T cell count. It remains to be investigated whether HIV patients with a severe suppression in T cell immunity are also able to respond to immune stimulators or whether virological suppression first has to be prioritized to enable T cell responsiveness.

### Targeted Strategies

The delivery system is also an important part of an immune-based strategy and implementation of various novel approaches based on liposomes, electroporation, dendrimers, carbon nanotubes, etc., can boost efficacy (92). For instance, as an alternative for broad cytokine adjuvants, more effective and tolerable approaches are being explored like encapsulation in micro or nanoparticles, restricting the delivery to APCs and/or the co-delivery with another immunomodulatory molecule *via* transducing vectors. Similar techniques such as microRNA or small interference RNA-based therapy could be explored, but these novel drugs will be most likely unaffordable in most countries where the disease is endemic.

### Accesibility

The target population is largely living in very rural and/or poor areas, where a highly controlled clinical trial setting can be challenging and costly to implement. It will be imperative to strengthen human and infrastructural capacity in disease endemic areas to ensure a sustainable base for immunotherapeutic research and to assess safety and efficacy of novel interventions. Moreover, designed therapeutics should become affordable and accessible to the patient population, suggesting innovative low-resource-demanding methods ideally without the need of a cold chain.

### Conclusion

To advance the development of immunomodulatory approaches for VL–HIV coinfection, a more detailed account of the immunological status induced by the coinfection and surrogate markers of cure and protection are still required, as a forerunner to inclusion of such patients in clinical intervention studies. The main limitation for comprehensive immunological research is, however, the need for human samples of longitudinal studies and trials in (often very remote) low-resource settings. With more research aimed at discovering key synergistic pathways of immune cell cross-talk and renewed efforts to translate these findings into effective treatment modalities that target *Leishmania* without promoting HIV replication, the goal of improved patient outcome and clinical management of this neglected population may be achievable.

## Author Contributions

WA, JG, GV, LK, TD, and PK wrote and conceived the review.

## Conflict of Interest Statement

The authors declare that the research was conducted in the absence of any commercial or financial relationships that could be construed as a potential conflict of interest.
